# Internet use and need for digital health technology among the elderly: a cross-sectional survey in China

**DOI:** 10.1186/s12889-020-09448-0

**Published:** 2020-09-11

**Authors:** Xinran Sun, Wenxin Yan, Hao Zhou, Zhaoqing Wang, Xueying Zhang, Shuang Huang, Li Li

**Affiliations:** 1 School of Health Management, Harbin Medical University, Harbin, China; 2 Department of Quality Control, Center for Disease Control and Prevention, Harbin, China

**Keywords:** Elderly, Internet use, Digital health technologies, Influential factor

## Abstract

**Background:**

China is becoming an aging society at the fastest pace in history, and there are a large number of empty nesters in the country. With economic and social development, internal support systems among families are gradually weakening. Supporting the elderly is thus emerging as a significant issue, and promoting digital health technologies is an effective way to help address it. Encouraging the application of Internet to elderly care and Internet use among the elderly are important means of promoting digital health technologies. This paper examines the current state of the use of the Internet by the elderly and factors influencing it (including physical, psychological, and social) as well as demand among the elderly for smart services.

**Methods:**

A total of 669 subjects over the age of 60 years were randomly selected from 13 cities in Heilongjiang province and surveyed using questionnaires from May 1 to July 31, 2018. The questionnaires were collected for descriptive statistics, the chi-square test, and the analysis of influential factors.

**Results:**

Of the people surveyed, 38.6% used the Internet. Their favorite online activity was online dating (74.2%), and the health information they obtained through the Internet was mainly related to diet (63.1%) and exercise (47.1%). The subjects demanded smart bracelets (MD = 2.80) and emergency callers (MD = 2.77). Gender, age, education, monthly income, quality of life, number of friends, and social participation were found to have an impact on Internet use.

**Conclusions:**

More measures are needed to reduce barriers to the use of the Internet and promote digital health technologies. The society, equipment manufacturers, and family members of the elderly should work together to enable them to reap the benefits of online technologies.

## Background

With the growth of the economy and improvements to medical services in China, the average life expectancy has increased. China is becoming an aging society at the fastest pace in history. In 2018, there were 166.58 million people in the country aged 65 or older [1]. People above the age of 65 are projected to represent 26% of China’s population, and those aged 80 and older are expected to represent 5%, by 2050 according to a report by the World Bank [2]. There are thus a large number of empty nesters in China. Elderly people living alone account for nearly 10% of the total, and those living with only spouses account for 41.9% according to the China Family Development Report in 2015 [3]. The traditional family-based model of support for the aged has thus significantly deteriorated.

In light of the rapidly aging population, the demand for care and health services for the elderly is growing. Against the backdrop of limited capacity and weakening of family-based care, developing digital health technologies has emerged as a solution to this problem. Policies advocating digital health technologies have been introduced in the last several years. In 2015, the Chinese government set itself the goal of “promoting the development of the digital health technologies’ industry.” [4] To promote the use of Internet for care for the elderly, it is important to improve their access to the Internet.

A number of older people have kept abreast of technological developments in recent years. A study in Spain showed that older adults were interested in learning and acquiring Information and Communication Technologies’ skills [5]. Xie found that elderly people had good knowledge of the Internet, especially because of its convenience and usefulness [6].

By the end of 2018, 98.6% of Internet users accessed it using mobile phones in China. A total of 48 and 35.9% used desktop and laptop computers to access the Internet, respectively, whereas 31.1% used TVs. The Internet penetration rate in China was 57.7%, and that in Heilongjiang Province was 52%. It ranked 18th of the country’s 31 provinces. However, many elderly believe that they are excluded from the online environment. By December 2017, Internet users aged 60 and older accounted for 5.2% of all Internet users and only 16.7% of the total population of the elderly [7]. The barriers they face to using the Internet included cost, inappropriate design, physical and mental limitations, mistrust, and a lack of time [8, 9]. For most elderly people in China, the Internet is a novelty that appeared when they entered middle or old age. It is thus challenging for them learn to use it [10].

Most studies in the area have focused on the impact of Internet use by the elderly as well as factors influencing it.

The demand among the elderly for the use of the Internet is relatively simple. Most of them use it for entertainment (such as listening to the music and watching videos), accessing information (such as news, stock market information, and health-related information), and communicating with others (such as use WeChat or Facebook) [11, 12]. Compared with other age groups, the elderly are primarily interested in the Internet for health reasons [13]. They are more likely to search for health information online [14]. Most elderly people learn how to use the Internet through their children or other young people, while a minority learns by itself.11 Regarding the frequency of Internet use, a study found that 47.4% of older adults in the U.S. spend more than 7 h per week on the Internet, and only 7.1% reported spending no time on it [15]. Wei’s study showed that the elderly in China who use the Internet every day of the week accounted for 48.5% of the total elderly population [16].

The influence of the Internet on the lives of the elderly as well as their physical and psychological health has attracted the attention of many scholars. Elderly people with health problems are especially likely to benefit from using the Internet because it allows them to carry out an increasingly diverse array of tasks [17]. A study has shown when the elderly have access to larger social networks, the risk of such diseases as hypertension significantly decreases [18]. The Internet can also serve them as a powerful tool for communication to meet their need for interpersonal and social interactions [19]. Higher levels of Internet use are significant predictors of higher levels of social support, reduced loneliness, better life satisfaction, and psychological well-being among the elderly [20]. Some researchers reported that older adults who successfully created and shared content online enhanced their social connections and reduced social isolation. Ding claimed that new media can be an important channel to promote and enhance the social adaptation of the elderly [21]. However, the impact of the Internet on different populations is different, and there are some drawbacks to Internet use. A survey of the online activities of 4113 American adults found that social isolation deepened with increasing activities online [22]. A study on teenagers found that Internet use may render them unable to distinguish between the Internet and reality, and make them more susceptible to cyberbullying [23]. The adverse effects of using the Internet on the elderly are more clearly manifested in their physical health. For example, sitting for a long time affects their blood circulation, leads to cardiovascular diseases, and causes cervical pain, joint pain, tinnitus, and dizziness [24].

Many studies have found that certain socioeconomic and demographic factors, including age, sex, education, income status, health literacy, and urban and rural conditions, are associated Internet use by the elderly [25–27]. Older adults who are male, younger, live in urban areas, and have higher income, education, and health literacy have a higher probability of Internet use [28, 29].

Some physical and functional health problems can pose barriers to Internet use [30]. The elderly often suffer from reduced vision and physical disabilities that make it more difficult for them to use the Internet [31]. They tend to have poor memory as well, and thus are slower at learning new procedures for the Internet [8].

Some studies have found that psychological factors, such as loneliness, personality, and perspectives on life, affect Internet use. Using the Internet is an efficient way to promote friendships and social interaction, and to avoid negative feelings related to loneliness [32]. In addition, Peng found that elderly people who are optimistic are more likely to use the Internet, as are those who think that they have the right to control their own lives [33]. Erikson and Johnson found that Internet use and self-sufficiency are significantly related, where such elderly people were more likely to use the Internet [34].

Social factors are also important aspects of Internet use by the elderly. Studies have suggested that elderly people with good social support have higher motivation to learn the Internet and tend to do so. Care and support by family, and their encouragement can significantly increase Internet use among the elderly [35]. Participation in activities with family and friends is also likely to increase the need and perceived usefulness of Internet connectivity to maintain social integration and ties [27].

This study describes the current state of Internet use among the elderly, the demand for digital health technologies, and factors affecting their online behavior. The results are important for improving Internet use among the elderly. On the one hand, they can provide a fundamental theory for the development of the service industry for the elderly, and on the other, they can yield valuable suggestions for meeting their needs, improving their quality of life, and enabling them to benefit from the Internet.

## Method

### Data and sample

The participants of this study were chosen from all cities of Heilongjiang Province, The internet penetration rate in Heilongjiang Province is 52%, considered a middling level in China [1]. Subjects were included in the study if they met the following criteria: aged 60 years or older, lucid, and competent at verbal communication. Participation in the survey was voluntary, and the participants were told that returning the questionnaires represented their informed consent.

### Data collection

A cross-sectional survey was conducted from May 1 to July 31, 2018. First, those aged 60 years and older who were willing and able to answer the questions were included in our sample. Second, participants were solicited from all 13 cities in Heilongjiang. Three communities in urban areas were randomly selected from each city according to certain economic factors. The data were collected through in-person interviews using a structured questionnaire. The interviews were conducted by trained graduate students from Harbin Medical University. A manual was created to provide suggestions on how to ask each question. Moreover, a pre-investigation was conducted to identify problems and provide further training to the interviewers. In total, 780 questionnaires were distributed. Participants who did not respond to the survey or did not answer questions about their Internet use were excluded. Thus, 669 valid questionnaires were collected, and the rate of effective recovery was 85.77%.

### Assessment tools

This study used a questionnaire composed of four sections.

- Section 1 focused on the respondents’ socioeconomic and demographic characteristics (see Supplementary file 1).
- Section 2 surveyed the situation of older adults using the Internet. First, the participants were asked: “Do you use the Internet?” Second, we recorded the frequency of their daily and weekly Internet use. Third, the participants were asked to describe the types of online activities and health information they had sought online. These two questions were multiple choice (see Supplementary file 1).
- Section 3 assessed the need for digital health technologies for older adults. The participants were asked: “What are your demands for digital health technologies in the following list?” The list included the smart bracelet, emergency caller, telemedicine, online health consultation, online appointment registration, and paying for medical expenses online. The respondents were asked to indicate the strength of their need on a five-point scale, ranging from 1 (not at all) to 5 (significantly needed). The scores were averaged across items to form a scale (see Supplementary file 1).
- Section 4 assessed factors affecting Internet use. The physiological factors included whether the participant had a chronic disease, and quality of life. The psychological factor was loneliness, and social factors included the number of friends and frequency of participation in social activities (see Supplementary file 1).

The EQ-5D scale was used to assess the participants’ quality of life. It was developed by the UK’s EuroQol Group to provide a simple and universal method for measuring health for clinical and economic evaluation. It contained five dimension: mobility, self-care, usual activities, pain/discomfort, and anxiety/depression [36, 37]. The rights holder is Bianca Smit who granted permission for us.

Each dimension consists of five levels: no difficulty, some difficulty, moderate difficulty, extreme difficulty, and not applicable.

The UCLA-20 Loneliness scale prepared by Hays et al. in 1987 was used to assess loneliness [38]. The ULS-8 Loneliness scale consists of eight entries, including six forward entries and two reverse entries. To render scores of the scales consistent, we converted them into reverse entries. The higher the total score was, the more lonely the relevant participant was determined to be. Each entry uses a four-level rating from 1 to 4 representing “never,” “rarely,” “sometimes,” and “always,” respectively. The total score ranged from 8 to 32 points. The scale was reasonably reliable in our sample with a value of Cronbach’s α of 0.77.

### Data analysis

The data were processed using Epidata, and were double-entered to ensure quality. Characteristics of the questionnaires were analyzed by using SPSS V.19.0. Statistics were reported for socioeconomic and demographic characteristics, the state of the elderly using the Internet, and the need for smart medical care. The mean differences were examined using t-tests, and categorical variable differences were examined using χ^2^ tests with the significance set to *p* < 0.05. Factors influencing Internet use were analyzed by using logistic regression, set at *p* < 0.05. In this study, the outcome variable was access to the Internet (0 for “does not use the Internet” and 1 for “uses the Internet”).

## Results

### Socioeconomic and demographic characteristics of respondents

Table 1 shows the socioeconomic and demographic status of the respondents. More than half the participants were female (65.3%), aged from 60 to 69 years (56.2%). Most participants were married (75.5%), and only 14.2% had a monthly income over ¥3000. In terms of education, most had completed secondary education or below (89.9%). The majority of respondents owned a house (77%) and lived with children or others (89.5%). A total of 78.9% suffered from chronic diseases.

**Table 1:** Socioeconomic and demographic characteristics of the respondents (*N* = 669)

Variables	Total (669)	Internet users (258)	
	n_1_	n_2_	%
Sex			
Male	232	75	32.3
Female	437	183	41.9
Χ^2^		5.832*	
Age			
60–69	376	193	51.3
70–79	228	62	27.2
≥ 80	65	3	4.6
Mean ± SD	69.23 ± 6.90	66.00 ± 5.03	
Χ^2^		69.925**	
Education			
Primary school or below	224	31	13.8
Secondary education	377	186	49.3
University degree	68	41	60.3
Χ^2^		89.816**	
Marriage status			
Single/widowed/divorced	164	46	28.0
Married	505	212	42.0
Χ^2^		10.141*	
Living arrangements			
Alone	63	27	42.9
With children or others	606	231	38.1
Χ^2^		0.541	
Monthly income (¥)			
< 1500	177	43	24.3
1500–2999	397	165	41.6
≥ 3000	95	50	52.6
Χ^2^		24.654**	
House as property			
Yes	515	220	42.7
No	154	38	24.7
Χ^2^		16.290**	
Chronic diseases			
Yes	481	189	39.3
No	188	69	36.7
Χ^2^		0.383	

*p < 0.05;**p < 0.01

The percentage of Internet users = n_2_/n_1_

### Univariate analysis of internet use by elderly

The results of chi-square tests are shown in Table 1. Internet use varied among the respondents by sex (*p* < 0.05), age (*p* < 0.01), education (*p* < 0.01), marital status (*p* < 0.05), monthly income (*p* < 0.01), and ownership of a house (*p* < 0.01). Internet use was more frequent among females than among males. A higher percentage of participants below 70 years of age and married used the Internet than those above 70 with some other marital status. The percentage of participants with high income and education, and who owned property used the Internet more frequently than those who had low income and education, and did not own property.

### State of internet use among elderly

As shown in Table 2, 38.6% of the respondents used the Internet. Approximately half (53.9%) reported spending less than 2 h per day online, and more than two-thirds (75.2%) went online 5–7 days a week. Chatting online (74.2%) was the favorite activity among them, followed by reading the news (59%), and watching films and listening to music (32.7%). Shopping (6.8%) was least favorite activity. The participants reported mainly searching for information on diet care (63.1%) and fitness (47.1%). Only 13.1% of them learned about disease-related information online.

**Table 2:** State of Internet use by the elderly (*N* = 258)

Variables	n	%
Number of Internet users	258	38.6
Daily online time		
< 2 h	139	53.9
2–5 h	91	35.3
> 5 h	28	10.9
Weekly online days		
< 3 days	27	10.5
3–5 days	37	14.3
5–7 days	194	75.2
Internet activity^a^		
Chatting online	190	74.2
Reading news	151	59.0
Watching videos and listening to music	81	32.7
Searching for health information	56	22.5
Playing games	37	15.0
Shopping	17	6.8
Specific health information content^a^		
Diet care knowledge	159	63.1
Fitness knowledge	113	47.1
Medication condition	75	29.8
Food safety news	72	28.6
Disease-related information	33	13.1

^a^Multiple-choice questions

### Demand for digital health technologies among the elderly

As shown in Table 3, the greatest demand among the elderly was for smart bracelets (M = 2.80), followed by emergency callers (M = 2.77), telemedicine (M = 2.63), and online health consultation (M = 2.58). By contrast, demand for online appointment registration (M = 2.52) and online payment of medical expenses was low (M = 2.45).

**Table 3:** Demand for digital health technologies

Service items	Range	M ± SD^a^
Smart bracelet	1–5	2.80 ± 1.07
Emergency caller	1–5	2.77 ± 1.01
Telemedicine	1–5	2.63 ± 0.94
Online health consultation	1–5	2.58 ± 0.95
Online appointment registration	1–5	2.52 ± 1.00
Pay for medical expenses online	1–5	2.45 ± 0.87

^a^*M* Mean, *SD* Standard deviation

Assignment description: The numbers 1–5 represent the range of responses from “not at all” to “significantly needed.”

### Factors influencing internet use

Table 4 presents the results of a binary logistic regression model to show predictors of Internet use. Age, sex, marital status, level of education, monthly income, quality of life, number of friends, and social participation were significantly associated with Internet use.

**Table 4:** Factors influencing Internet use

Variables	B	*P* values	OR	95% CI	
Sex (ref = Female)	0.659	0.003	1.933	1.260	2.695
Age	−0.119	0.000	0.888	0.851	0.926
Education (ref = Primary school or below)		0.000			
Secondary education	1.279	0.000	3.591	2.141	6.024
University degree	1.943	0.000	6.978	3.080	15.806
Marriage status (ref = Married)	0.332	0.269	1.393	0.774	2.507
Monthly income (¥) (ref ≥ 3000)		0.035			
< 1500	−0.987	0.011	0.373	0.174	0.800
1500–3000	−0.485	0.122	0.616	0.333	1.138
Number of children	−0.134	0.230	0.874	0.703	1.088
Chronic diseases (ref = No)	−0.353	0.117	1.423	0.916	2.210
House property (ref = No)	−0.030	0.911	0.970	0.572	1.645
Living arrangements (ref = With children or others)	−0.697	0.078	0.498	0.229	1.082
Quality of life	2.241	0.036	9.404	1.161	76.176
Loneliness	−0.031	0.263	0.969	0.917	1.024
Social participation	0.255	0.025	1.290	1.032	1.614
Number of friends	0.262	0.021	1.299	1.040	1.623

B: Regression coefficient

*CI* CI code, *OR* ratio of odds

Compared with males, females (OR 1.933, 95% CI 1.260 to 2.695, *p* < 0.05) were more likely to use the Internet. The likelihood of the elderly using the Internet declined with age (OR 0.888, 95% CI 0.851 to 0.926, *p* < 0.001). The odds rations show that secondary education and a university degree rendered participants 3.6 times and seven times more likely to use the Internet, respectively, than those with primary school education or lower (OR 3.591, 95% CI 2.141 to 6.024, *p* < 0.001 and OR 6.978, 95% CI 3.080 to 15.806, *p* < 0.001, respectively). We also found those with income under ¥1500 were less likely to use Internet (OR 0.373, 95% CI 0.174 to 0.800, *p* < 0.05). When the quality of life, social participation, and number of friends increased by a grade, Internet usage increased by 2.241 (OR 9.404, 95% CI 1.161 to 76.176, *p* < 0.05), 0.255 (OR 1.290, 95% CI 1.032 to 1.614, *p* < 0.05), and 0.262 (OR 1.299, 95% CI 1.040 to 1.623, *p* < 0.05), respectively.

## Discussion

This study investigated the current state of Internet use among the elderly in China and factors that influence it. The results can provide theoretical and practical guidance for society, equipment manufacturers, and family members of elderly people to promote the use of the Internet among the elderly.

First, we examined the state of Internet usage among the elderly (Table 2). A total of 38.6% seniors used the Internet. Compared with other developed regions in China and people of other age groups, this ratio was low [39–41]. The popularity of the Internet is significantly affected by the level of economic development [42]. Because of the low economic development in Heilongjiang province, the number of elderly people using the Internet was small. In terms of the frequency of the use of the Internet, we found that more than half the elderly respondents surveyed used the Internet for less than 2 h every day and 5 to 7 days every week. The findings indicated that although the elderly used the Internet for short periods of time, they used it almost every day.

Second, we analyzed activities of the elderly using the Internet (Table 2). The Internet can offer a diverse array of such activities, and can help them maintain social relations and reduce the feeling of social isolation. Consistent with previous observations, chatting online (74.2%) was the favorite online activity among them [43]. The Internet provides a convenient way to communicate with their families, friends, and others, regardless of temporal and geographical restrictions [44]. We also found that the elderly liked to browse the news (59%), and watch films and listen to music online (32.7%). This allowed them to obtain information related to their hobbies at any time and attend to current events [45].

With aging and the loss of physiological functions, the elderly are eager to obtain health-related knowledge. The Internet contains rich and comprehensive health information that can meet their demands to a certain extent, and this increases the frequency with which the elderly use the Internet. A study of elderly such as African- Americans, Hispanics and others showed that 63% of the elderly had used the Internet to obtain health-related information, a higher percentage than for other activities, such as paying bills or ordering a product [46]. The results revealed that 22.5% of them searched for health information on the Internet, including information about food, drugs, and diseases. They focused on dietary knowledge and knowledge of fitness (63.1 and 47.1%, respectively). They also applied this knowledge to health management. However, note that there is a large amount of unscientific or incorrect health-related information on the Internet that can mislead the elderly.

The development of the Internet has changed the way people shop. We discovered that online shopping was the least favorite activity among the elderly (6.8%). There are two reasons for this. First, they lack the sufficient trust in Internet security systems and fear that their personal information may be leaked. Second, the elderly are not proficient enough in the relevant operation of the Internet to shop [11].

Third, we examined the demands of the elderly for digital health technologies (Table 3). We found that their demands were at a medium level (Table 3). The highest demand was reported for smart bracelets (M = 2.80), a mobile health-monitoring tool that can automatically collect data on the user’s activities and health, such as quality of sleep and heart rate. The elderly also demanded emergency callers (M = 2.77) for assistance in emergencies, such as sudden illness or accidents. They also wanted telemedicine services (M = 2.63). These products provide basic health management and medical services for the elderly. Their use is mainly influenced by demand from suppliers and the elderly. From the government’s point of view, it is possible to establish colleges for seniors to provide the necessary support and assistance [35]. Manufacturers should also make products more suitable for the elderly and provide technical support. Family members of the elderly should also support, encourage, and help them learn and use these products [47]. At the same time, the elderly should cultivate the skills of active learning.

Finally, we examined factors influencing Internet use among the elderly. This can help understand the difficulties and challenges faced by them in using the Internet.

Our results suggested that among demographic factors, being a female, and higher education and income had a significant impact on Internet use among the elderly (Table 4). Greater usage with higher education and income was also consistent with previous studies [48]. Income status is an important reference factor for measuring the capability of the elderly for online consumption. A lack of sufficient financial support directly affected purchasing power, which restricted the use of Internet. Thus, smart product manufacturers should develop items that are affordable and easy to operate for the elderly.

The results showed that quality of life positively contributed to online activity (Table 4). This finding is consistent with numerous studies [49, 50]. We found that age was significantly associated with Internet use (Table 4). Most elderly people suffer some decline in physical, cognitive, visual, aural, and other functions with age, which may be a barrier to Internet use [51]. In addition, when the quality of life increased by a grade, Internet use increased 2.241 times. The quality of life is a multi-dimensional concept that includes not only physical health, but also psychological health and social health. The psychological health of the elderly and their use of the Internet can affect each other. The elderly are also prone to such psychological problems as loneliness and fear, which enhances their tendency to use the Internet. On the contrary, Internet use can also improve their psychological health [32, 52].

Social participation by the elderly and the number of friends they have are important reflectors of their quality of life. Internet used by the elderly was significantly associated with their number of friends and social participation. Our data showed that when the number of friends and social participation of a respondent increased by a grade, Internet use increased by 0.262 and 0.255, respectively (Table 4), and this is consistent with previous findings [47]. The influence of peer group was also an important environmental factor. Furthermore, the elderly were more likely to be motivated to learn and use the Internet through peer support and encouragement.

## Limitations

Two limitations of this study should be noted. First, it was based on a small sample of the elderly population, which limits the generalizability of the research findings here. A survey was conducted in 13 cities (regions) in Heilongjiang Province to ensure that the data were representative. Second, this study focused on whether the elderly use the Internet without an extensive analysis of how they use it.

## Conclusion

This study investigated the state of Internet use and demand for digital health technologies among the elderly, and studied factors influencing their online activity. The results show that 38.6% of the respondents used the Internet. Factors influencing Internet use among the elderly were sex, age, education, income, quality of life, social participation, and number of friends. In the future, society, equipment manufacturers, and family members of elderly people should work together to mitigate barriers to Internet use for them. Greater social interaction among them should also be encouraged to enable them to reap the benefits of the information age.

## Supplementary information

**Additional file 1: Supplementary file 1.** Questionnaire for the elderly and the demand for digital health technology in Heilongjiang Province

## Data Availability

The datasets generated and/or analyzed during the current study are available from the corresponding author on reasonable request.

## Abbreviations

EQ-5D: EuroQol Five-Dimensions Questionnaire
UCLA: University of California at Los Angels
ULS-8: The short-form of the UCLA Loneliness Scale
SPSS: Statistical Product and Service Solution
M: Mean
SD: Standard deviation
B: Regression Coefficient
CI: Confidence Interval
OR: Ratio of odds

## References

[1] National Bureau of Statistics http://data.stats.gov.cn/easyquery.htm?cn=C01&zb=A0301&sj=2018. Accessed 8 Oct 2018.

[2] IBRD.IDA. Options for aged care in China: Building an efficient and sustainable Aged care system (English). http://www.shihang.org/zh/news/press-release/2018/12/13/world-bank-report-offers-options-for-elderly-care-in-china .Accessed 13 Dec 2018.

[3] National Health and Family Planning Commission Family Division (2015). China Family Development Report (2015).

[4] National Health Commission of the people’s republic of China http://www.nhc.gov.cn/cms-search/xxgk/getManuscriptXxgk.htm?id=9b032c93f6a94d7fb80b832155ea8d89. Accessed 16 Dec 2017.

[5] Padilla-Góngora D, López-Liria R, del Pilar D-LM (2017). Habits of the elderly regarding access to the new information and communication technologies. Procedia Soc Behav Sci.

[6] Xie XL. The characteristic of old adults’ use of the internet and its associations with their loneliness and subjective well-being [D]. Beijing: Central China Normal University; 2015.

[7] China Internet Network Information Center. The 43rd Statistical Report on the Development of China’s Internet. http://cnnic.cn/gywm/xwzx/rdxw/20172017_7056/201902/t20190228_70643.htm. Accessed 28 Feb 2019.

[8] Gatto SL, Tak SH (2008). Computer, internet, and E-mail use among older adults: benefits and barriers. Educ Gerontol.

[9] He Q, Zhang XD (2017). The influence factors of the digital divide in the elderly and the social integration strategy. J Zhejiang Univ Technol (Soc Sci).

[10] Chen K. An encounter between the aged and new media: AnaIyzing the media life under the perspective of positive aging based on the research of new media classroom in Hefei [D]. Hefei: Anhui University; 2017.

[11] Loipha S (2014). Thai elderly behavior of internet use. Procedia Soc Behav Sci.

[12] Zhou J, Rau PLP, Salvendy G (2014). Older adults’ use of smart phones: an investigation of the factors influencing the acceptance of new functions. Behav Inform Technol.

[13] Salovaara A, Lehmuskallio A, Hedman L (2010). Information technologies and transitions in the lives of 55–65-year-olds: The case of colliding life interests. Int J Human Comp Stud.

[14] Zhu SB, Deng XZ (2015). Study on influence factors of older health information seeking. Libr Inform Serv.

[15] Zheng R, Spears J, Luptak M (2015). Understanding older adults’ perceptions of internet use: an exploratory factor analysis. Educ Gerontol.

[16] Wei DW. Research about the obstacles of use of the internet to the elderly in China from the perspective of the digital divide [D]. Wuhan: Wuhan Textile University; 2012.

[17] Xie B (2011). Older adults, e-health literacy, and collaborative learning: an experimental study. J Assoc Inform Sci Technol.

[18] Cornwell EY, Waite LJ (2012). Social network resources and management of hypertension. J Health Soc Behav.

[19] Leeson GW. The Comparison in the Effect of Use of Internet and Social Support on the Depression of the Urban Elderly: Evidence from the Sample Survey of Three Cities in West China. Special Zone Econ Issue. 2018(009):89–91.

[20] Heo J, Chun S, Lee S (2015). Internet use and well-being in older adults. Cyberpsychol Behav Soc Netw.

[21] Ding ZJ, Shen Q (2017). The influence of new media on the social adaptation of urban elderly. Contemp Commun.

[22] Cotten SR, Anderson WA, Mccullough BM (2012). The impact of ICT use on loneliness and contact with others among older adults. Gerontechnology.

[23] Singh S, Thapar V, Bagga S (2020). Exploring the hidden patterns of cyberbullying on social media. Procedia Comp Sci.

[24] Wei-Yang Z (2018). The Elderly Use Internet Behavior Research. education modernization.

[25] Slegers K, Boxtel MPJV, Jolles J (2012). Computer use in older adults: determinants and the relationship with cognitive change over a 6 year episode. Comput Hum Behav.

[26] Zhang S (2013). The factors affecting the old people’s use of new medias in China’s urban areas:a survey in Chaoyang District of Beijing. South China Popul.

[27] Choi NG, Dinitto DM, Eysenbach G (2013). Internet use among older adults: association with health needs, psychological capital, and social capital. J Med Internet Res.

[28] Levy H, Janke AT, Langa KM (2015). Health literacy and the digital divide among older Americans. J Gen Intern Med.

[29] Wu XH (2017). Internet use and its influence on the elderly: A study based on CSS (2013) questionnaire data analysis. J Yunnan Minzu Univ (Social Sciences).

[30] Medeiros FL, Xavier AJ, Schneider IJ (2012). Digital inclusion and functional capacity of older adults living in Florianópolis, Santa Catarina, Brazil (EpiFloripa 2009-2010). Revista Brasileira De Epidemiologia.

[31] Wagner N, Hassanein K, Head M (2010). Computer use by older adults: a multi-disciplinary review. Comput Hum Behav.

[32] Haydar A (2012). Is the Internet use an effective method to cope with elderly loneliness and decrease loneliness symptom?. Procedia Soc Behav Sci.

[33] Peng QY (2018). A study on the barriers of internet access for the urban elderly. Popul Econ.

[34] Erikson J, Johnson GM (2011). Internet use and psychological wellness during late adulthood. Can J Aging.

[35] Xie XL, Chen Y, Lao YX (2017). Status quo, influencing factors and coping strategies of internet use among the elderly. Chin J Gerontol.

[36] Rabin R, Charro FD (2001). EQ-SD: a measure of health status from the Europe group. Ann Med.

[37] Tsuchiya A, Ikeda S, Ikegami N (2002). Estimating an EQ-5D population value set:the case of Japan. Health Econ.

[38] Hays RD, Dimatteo MR (1987). A short-form measure of loneliness. Pers Assess.

[39] Lv XJ, Ding Y (2013). Analysis on demand willingness and influence factors of network pension service for the aged--based on the survey data of the Beijing demand will of urban elderly network pension. Soc Sec Stud.

[40] Bauer R, Glenn T, Strejilevich S (2018). Internet use by older adults with bipolar disorder: international survey results. Int J Bipolar Disord.

[41] Barbara BN, Fonseca JRS, Fausto A (2018). Social capital and Internet use in an age-comparative perspective with a focus on later life. PloS One.

[42] Wang M, Wang QM, Wan B (2018). Spatial difference of internet popularization in China and analysis on its influencing factors. Stat Decis.

[43] Maria CR, Lauren MK, Marian R (2018). Mobile phone, computer and internet use among older homeless adults: results from the HOPE HOME cohort study. JMIR MHealth UHealth.

[44] Choi NG, DiNitto DM (2013). The digital divide among low-income homebound older adults: internet use patterns, eHealth literacy, and attitudes toward computer/internet use. J Med Internet Res.

[45] Mellor D, Firth L, Moore K (2008). Can the internet improve the well-being of the elderly?. Ageing Int.

[46] Gordon NP, Hornbrook MC (2018). Older adults’ readiness to engage with eHealth patient education and self-care resources: a cross-sectional survey. BMC Health Serv Res.

[47] Xie LL (2014). Study on the factors influencing the elderly’s internet usage based on the planned behavior theory. Sci Res Aging.

[48] Choi N (2011). Relationship between health service use and health information technology use among older adults: analysis of the National Health Interview Survey. J Med Internet Res.

[49] Hajek A, König H-H (2019). The association between use of online social networks sites and perceived social isolation among individuals in the second half of life: results based on a nationally representative sample in Germany. BMC Public Health.

[50] Levine DM, Lipsitz SR, Linder JA. Changes in everyday and digital health technology use among seniors in declining health. J Gerontol. 2017(4):552–9. 10.1093/gerona/glx116 28605446

[51] Hussain D, Ross P, Bednar P (2018). The perception of the benefits and drawbacks of internet usage by the elderly people.

[52] Hong JZ, Huang F, Pi ZL (2015). Internet use and mental health of the elderly. J Central China Normal Univ (Humanities Soc Sci).

