# Supplementary material for: Internet use and need for digital health technology among the elderly: a cross-sectional survey in China
Source: BMC Public Health. 2020 Sep 11;20:1386. doi: 10.1186/s12889-020-09448-0 (PMC7488462; doi:10.1186/s12889-020-09448-0)
Supplement: Supplementary file 1 — Additional file 1: Supplementary file 1. Questionnaire for the elderly and the demand for digital health technology in Heilongjiang Province [file 12889_2020_9448_MOESM1_ESM.docx]

**Internet use and demand for digital health technology**

**among the elderly in Heilongjiang Province**

Thank you very much for reading and filling out this questionnaire during your busy schedule. We are conducting a survey on “Internet use and demand for digital health technology among the elderly in Heilongjiang Province”. The purpose of this survey is to understand Internet use status and demand for digital health technology among the elderly in Heilongjiang Province at this stage. The information you provide will provide an important reference for our study. This survey is completely voluntary, if you have any objections, you can withdraw at any time. And complete the questionnaire and return it to us indicate your informed consent to apply personal information to this research. The questionnaire is filled in anonymously and will not bring you any adverse effects and consequences. Please mark "√" on the corresponding option according to the actual situation; if you need to fill in the specific number, please fill in the number on the corresponding “ ”. We look forward to your valuable opinions! Thank you for your support and cooperation!

1. **Basic information.**

A1. Gender: (1) Male (2) Female

A2. Age: years old

A3. Education: (1) Primary school or below (2) Secondary education (3) University degree

A4. Current marriage status: (1) Married (2) Single (3) Widowed (4) Divorced

A5. Who do you live with? (1) Alone (2)With children or others

A6. Your monthly income is about Yuan

A7. Do you have your own house as property? (1) Yes (2) No

A8. Are you suffering from chronic diseases? (1) Yes (2) No

A9. how many children do you have?

A10. How many close friends do you have who can support and help you (excluding relatives and family members)?

(1)0 (2) l - 2 (3) 3 - 5 (4) > 5

A11. For groups organize activities (such as party organizations, religious organizations, unions, communities, etc.)

(l) Never participate (2) Occasional participation

(3) Often participate (4) Active participation

1. **Internet usage status.**

B1. Do you use the Internet? (1) yes (2) no

B2. How many hours do you use the Internet every day?

1. less than 2 hours (2) 2-5 hours (3) more than 5 hours

B3. How many days do you use the Internet every week?

1. Less than 3 days (2) 3-5 days (3) 5-7 days

B4. What types of online activities do you do through the Internet？(Multiple-choice questions)

1. Chatting online (2) Reading news (3) Watching videos and listening to music
2. Playing games (5) Shopping (6) Searching for health information

B5. What health information do you get through the Internet? (Multiple-choice questions)

1. Food safety news (2) Fitness knowledge (3) Medication condition
2. Diet care knowledge (5) Disease-related information
3. **Demand for digital health technologies. (Please tick “√”on the option)**

What are your demands for digital health technologies in the following list?

|  | not at all | no need | general | needed | significantly needed |
| --- | --- | --- | --- | --- | --- |
| Smart bracelet | 1 | 2 | 3 | 4 | 5 |
| Emergency caller | 1 | 2 | 3 | 4 | 5 |
| Telemedicine | 1 | 2 | 3 | 4 | 5 |
| Online health consultation | 1 | 2 | 3 | 4 | 5 |
| Online appointment registration | 1 | 2 | 3 | 4 | 5 |
| Pay for medical expenses online | 1 | 2 | 3 | 4 | 5 |
